# Combined TLR2 and TLR4 ligation in the context of bacterial or helminth extracts in human monocyte derived dendritic cells: molecular correlates for Th1/Th2 polarization

**DOI:** 10.1186/1471-2172-10-9

**Published:** 2009-02-04

**Authors:** Elly van Riet, Bart Everts, Kim Retra, Marion Phylipsen, Jaap J van Hellemond, Aloysius GM Tielens, Desiree van der Kleij, Franca C Hartgers, Maria Yazdanbakhsh

**Affiliations:** 1 Department of Parasitology, Leiden University Medical Center, Leiden, The Netherlands; 2 Department of Biochemistry and Cell Biology, Faculty of Veterinary Medicine, Utrecht University, Utrecht, The Netherlands; 3 Department of Medical Microbiology and Infectious Diseases, Erasmus MC University Medical Center, Rotterdam, The Netherlands; 4 TNO Defense, Security and Safety, Business Unit Biological and Chemical Protection, Rijswijk, The Netherlands; 5 Division of Drug Delivery Technology, Leiden Amsterdam Center for Drug Research (LACDR), Leiden, The Netherlands

## Abstract

**Background:**

Recognition of pathogens by dendritic cells (DCs) through interaction with pattern recognition receptors, including Toll like receptors (TLR), is crucial for the initiation of appropriate polarized T helper (Th) cell responses. Yet, the characteristics and differences in molecular profiles of DCs with different T cell polarizing capacities are still poorly defined. To address this issue, the molecular profile of human monocyte derived DCs was characterized after exposure to TLR4 ligand LPS in combination with the Th1 promoting bacterial extracts from *Listeria monocytogenes *and *Escherichia coli *or the Th2 promoting helminth derived phospholipids from *Schistosoma mansoni *and *Ascaris lumbricoides*, all with TLR2 activating capacity.

**Results:**

With regard to the signalling pathways activated upon exposure to LPS and the TLR2 activating compounds, we find that the ratio of activated Mitogen Activated Protein Kinases (MAPK) *p*-ERK/*p*-p38 is lower in DCs stimulated with the bacterial products compared to DCs stimulated with the helminth products, which correlates with the Th1 and Th2 polarizing capacity of these compounds. Furthermore, analysis of the mRNA expression levels of a set of 25 carefully selected genes potentially involved in modulation of T cell polarization revealed that the mRNA expression of notch ligand delta-4 and transcription factor c-fos are differentially regulated and show a strong correlation with Th1 and Th2 polarization, respectively.

**Conclusion:**

This study shows that combined TLR2 and TLR4 activation in the context of different antigen sources can induce very distinct molecular profiles in DCs and suggests that the Th1/Th2 polarizing capacity of compounds can be predicted with the molecular signature they induce in DCs.

## Background

Dendritic cells (DCs) are antigen presenting cells that play a pivotal role in the initiation of adaptive immune responses. These cells function as sentinels in the periphery where they are able to recognize and respond to stimuli from the environment they reside in, some of which could be products from invading micro-organisms or helminths. Upon such exposures DCs undergo phenotypic changes that allow them to effectively migrate to lymph nodes and prime appropriate T cell responses [1,2].

The type of compounds encountered by DCs will determine to a large extent the nature of the T cell polarization promoted by these DCs. For this, DCs have to be able to distinguish these different classes of molecules. To this end, DCs express several classes of pattern recognition receptors (PRR), such as Toll-like receptors (TLR), C-type lectin receptors, Nod-like receptors and RIG-I like receptors that are able to recognize specific pathogen derived components, the so-called pathogen associated molecular patterns (PAMP). Upon engagement of these receptors, signalling cascades are initiated that involve activation of the MAPK and Nuclear factor-κB (NF-κB), and induction of expression of genes involved in DC maturation and the ability to prime and skew T cell responses [3]. It is known that intracellular organisms are primarily capable of instructing DCs to induce Th1 responses [4], whereas extracts of parasitic helminths have been demonstrated to drive Th2 skewed responses [4-6].

Relatively much is known about the signalling pathways in DCs induced after triggering of PRR [3,7-9], however, the molecular characteristics that are different for DCs that have been activated by Th1 or Th2 promoting PAMP are much less understood [10,11]. We set out to address this issue by characterizing human monocyte derived DCs after exposure to maturation stimulus LPS, in combination with bacterial and helminth derived products. The characterization of the DCs comprised gene expression analysis of 25 genes that have been linked to activation and T cell polarizing properties of DCs. These molecular profiles of the DCs were correlated to their T cell polarizing capacity. In this study we used Gram-positive heat killed *Listeria monocytogenes *(HKLM) and Gram-negative *Escherichia coli*, both of which stimulate TLR2 and induce Th1 polarization. In addition, *Schistosoma mansoni *and *Ascaris lumbricoides *derived phosphatidylserine containing preparations (PS) were used, that also activate TLR2, but drive Th2 responses in the presence of TLR4 ligation by LPS [6]. We show that the signalling routes and the resulting mRNA expression profiles following stimulation by the bacterial and helminth derived products are very distinct. This indicates that not all extracts that contain TLR2 activating components modulate DC programming by LPS in a similar fashion and in addition suggests that there is a general molecular DC1 and DC2 signature that can be used to predict Th1 and Th2 skewing potential of DCs.

## Results

### TLR2 activating components that induce Th1 or Th2 polarization via DCs

**Figure 1:**
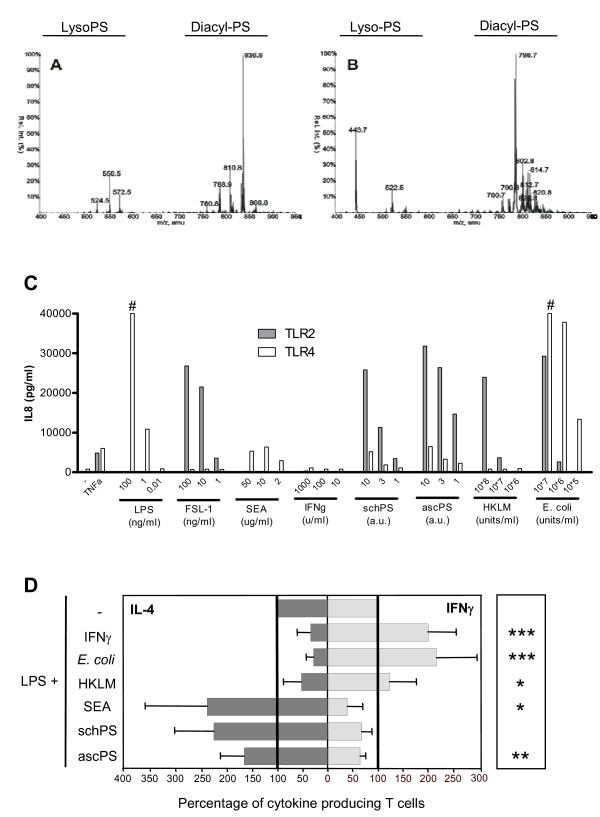
**TLR activation and T-cell polarization by the different compounds**. Mass spectrometry analysis of schPS (A) and ascPS (B). Samples were analysed by LC/MSMS in the negative mode. Neutral loss scans of 87 amu, corresponding to the loss of serine from the phospholipid were obtained. The relative intensity is shown of the detected phosphatidylserine species (indicated by their distinct m/z ratios). C. Activation of TLR2 and TLR4 transfected HEK293 cells. HEK cells were stimulated and IL-8 production in response to activation is shown. CD14 transfected HEK cells were used as negative controls (not shown). One representative experiment out of at least two independent experiments is shown, based on triplicate wells. # > 40.000 pg/ml. 1 a.u. is referring to lipids derived from 2 worm pairs/ml or 12 mg of worm/ml for SchPS and AscPS, respectively. D. T cell polarization was determined by measuring the percentages of cells with intracellular IFN-γ and IL-4 production by FACS analysis. T-cell polarization after LPS stimulation alone (4,6 ± 3,5% IL-4 and 33,9 ± 15,1% IFN-γ producing T cells, respectively) was set to 100% (indicated by the bold lines). Relative amounts of IFN-γ and IL-4 positive T cells induced by the stimuli in the presence of LPS are given. Dark gray (left); IL-4, Light grey (right); IFN-γ. Error bars represent SD of the mean of at least 4 independent experiments where cytokines produced in T cells of single wells of cocultures were measured. Significant differences in IL-4/IFN-γ ratio for the different conditions relative to the LPS control are depicted on the right side of the graph. * p < 0.05, ** p < 0.01, *** p < 0.001.

To study the molecular characteristics of DCs exposed to compounds that engage TLR2 and 4, yet lead to differential skewing of immune responses in terms of Th1 and Th2 induction, different pathogen derived products from bacterial or helminth origin with a known Th1 and Th2 inducing capacity were chosen and combined with LPS, as a reference maturation stimulus [6]. For this study Gram-negative *E. coli *and Gram-positive heat killed *L. monocytogenes *(HKLM) were used as bacterial stimuli that induce Th1 responses. A schistosome (a trematode) derived phosphatidylserine containing lipid preparation (schPS) and a similar preparation from the nematode worm *A. lumbricoides *(ascPS), both containing mainly phosphatidylserine species with two attached acyl chains and some lysophosphatidylserine species (with only a single attached acyl chain) (fig 1A and 1B, respectively), were chosen as Th2 inducing compounds [6]. Stimulation of HEK cells transfected with TLR showed that all stimuli could activate TLR2, with additional weak and potent TLR4 stimulation by the helminth lipids and *E. coli*, respectively (fig. 1C). IFN-γ and schistosome derived soluble egg antigen (SEA), stimuli that do not show TLR2 activating capacity in our experiments (fig. 1C), and induce Th1 and Th2 responses, respectively, were used as controls [6]. To assess the T cell polarizing capacity of DCs exposed to these compounds, stimulated human monocyte derived DCs were co-cultured for two weeks with allogeneic naïve CD4^+ ^T cells and IL-4 as well as IFN-γ production was determined by intracellular staining upon T cell restimulation (fig. 1D). DCs were stimulated with the different compounds in the presence of LPS, to ensure equal maturation and to rule out potential effects on polarization due to differences in maturation status of the DCs. We found that in all conditions expression of maturation markers was significantly higher than levels measured on immature DCs and more similar to the levels induced by LPS alone (data not shown). As expected, *E. coli *induced a strong Th1 response comparable to DCs stimulated with IFN-γ, while HKLM induced a moderately polarized Th1 response. Conversely, the helminth derived compounds, as shown before for schPS [6], and SEA [4,6], but also the *A. lumbricoides *derived phospholipids instructed DCs to drive Th2 skewed responses with the strongest polarization induced by SEA (fig. 1D). Based on intracellular IL-17 staining there was no sign of Th17 induction by the differently conditioned DCs, which is in agreement with other studies [12,13].

### MAPK activation

**Figure 2:**
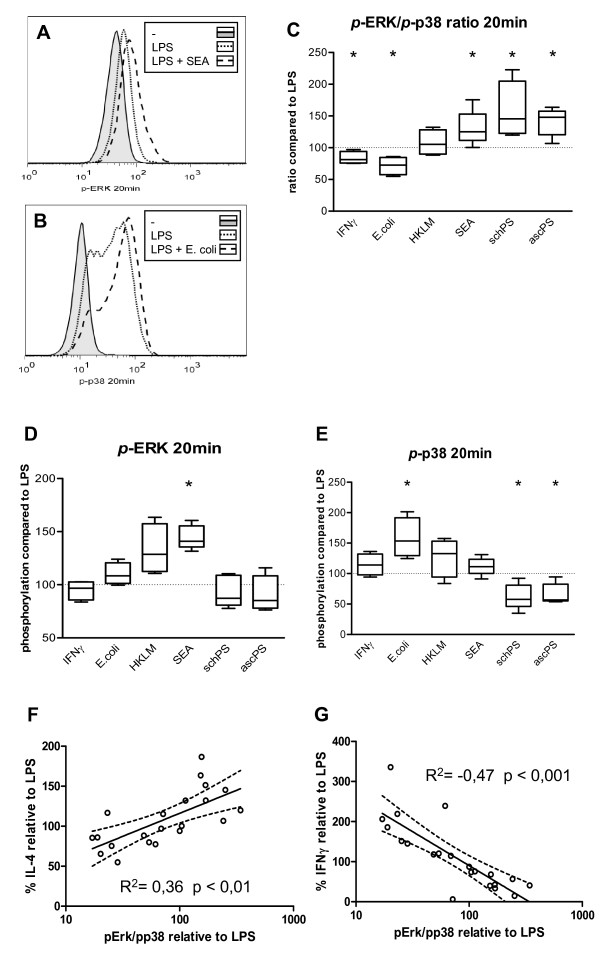
**MAPK activation in DCs**. Representative histograms of (A) ERK and (B) p38 phosphorylation in DCs 20 minutes after stimulation. C-E. Ratios of *p*-ERK/*p*-p38 (C), phosphorylation of ERK (D) and p38 (E) 20 minutes after stimulation in the presence of LPS. Expression induced by LPS (MFI of 49 ± 12 for *p*-ERK and 16 ± 9 for *p*-p38) was set to 100% (dashed line). Relative expression levels or ratios are shown. * P < 0.05 compared to LPS stimulation. F and G. Correlation of *p*-ERK/*p*-p38 ratio and IL-4 (F) or IFN-γ (G) production by T-cells. All data are relative to stimulation with LPS only and combined results from 4 independent experiments are shown.

To obtain a better understanding of the molecular processes in DCs that could underlie the observed differences in T cell polarizing capacity of these helminth- and bacteria-derived compounds, we set out to investigate in more detail the molecular characteristics of DCs exposed to the different stimuli. To study the intracellular signalling routes activated upon exposure to the helminth and bacterial derived products, we analysed the activation of the MAPK. ERK (ERK1/2) and p38 are two effector kinases of the MAPK family and are known to play an important role in shaping of immune responses [14]. p38 has been shown to regulate DC maturation and pro-inflammatory responses, while activation of ERK has been related to anti-inflammatory and Th2 responses [15]. As has been described before [16], exposure of DCs to LPS alone led to preferential phosphorylation of p38 (fig. 2A, B). The Th1 promoting stimuli IFN-γ and *E. coli *even further increased the activation of this MAPK resulting in a reduced *p*-ERK/*p*-p38 ratio, 20 minutes after stimulation (fig 2B, C), whereas for HKLM this ratio did not change. In contrast, the Th2 inducing compounds PS and SEA increased this ratio. Interestingly, the high *p*-ERK/*p*-p38 ratio induced by these Th2 polarizing stimuli was the result of different activation profiles for SEA versus the lipid preparations: SEA significantly induced phosphorylation of ERK, whereas the helminth derived lipids impaired p38 activation, but showed no effect on ERK activation (fig 2D and 2E). The *p*-ERK/*p*-p38 ratio showed a positive correlation with Th2, and negative correlation with Th1 polarization (R^2 ^= 0.36 and -0.47, respectively, figure 2F and 2G). In conclusion, for all components tested, the *p*-ERK/*p*-p38 ratio only 20 minutes after DC stimulation can be used to predict the outcome of the T cell response with regard to Th1 and Th2 polarization. This shows that very early events in DC activation already determine the fate of the DCs in terms of their T cell polarizing capacity.

### Gene expression analysis

**Figure 3:**
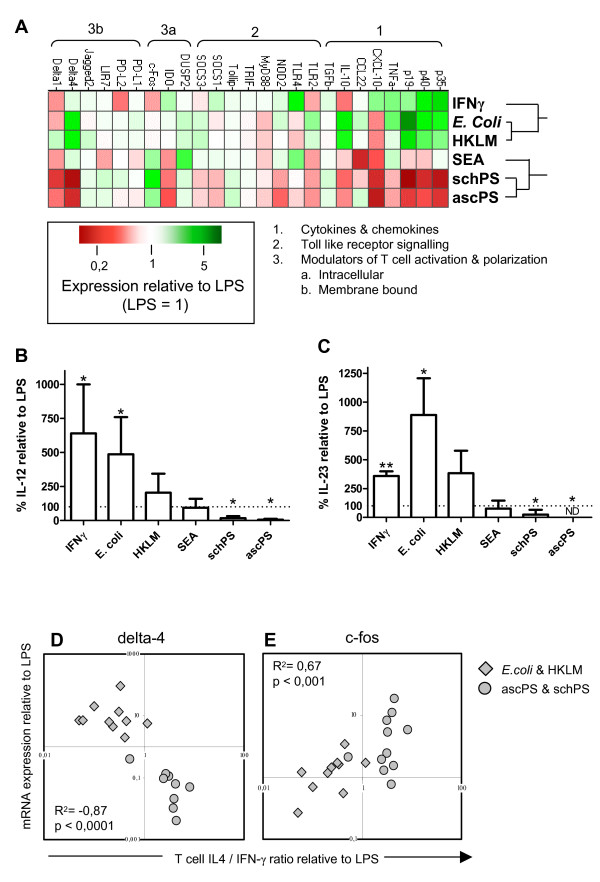
**Distinct mRNA expression levels after stimulation of immature DCs with Th1 and Th2 inducing compounds**. A. mRNA expression levels of the different genes compared to LPS (representing a value of 1). Green and red colours represent expression levels higher and lower, respectively, relative to LPS pulsed DCs. Expression was determined by real-time PCR with TAF-1 as housekeeping gene. Stimuli were clustered hierarchically according to expression profiles (top of figure). B and C. Amount of IL-12 p70 and IL-23 present in the supernatant of DCs 48 hours after stimulation, relative to the amount produced in the presence of LPS only (2518 ± 1733 pg/ml IL-12 & 230 ± 195 pg/ml IL-23). ND: not detectable, * p < 0.05, ** p < 0.01 compared to LPS stimulation only. D and E. Association of delta-4 (D) and c-fos (E) mRNA expression levels with T cell polarization for TLR2 activating stimuli. Diamonds represent HKLM or E. coli, circles the helminth derived lipids. Relative expression levels to LPS control condition (set to 1) from at least 3 independent experiments are shown.

To further characterize the molecular profile of the differentially stimulated DCs we performed mRNA expression analysis, using real-time PCR, on a selected set of genes involved in TLR signalling and T cell polarization (table 1[4,15,17-48], Figure 3A). Upon maturation with LPS, the expression of most genes was increased (data not shown). All data shown are relative to what is seen in mature DCs without any polarizing agents added, i.e. DCs stimulated with LPS. Stimulation of DCs from different individuals with the same stimulus showed very consistent profiles (data not shown). Clustering analysis revealed that the gene expression profiles of Th1 and Th2 polarizing agents clustered in separate groups (top of figure 3A). Within the Th1 stimuli, DCs exposed to bacterial products derived of *L. monocytogenes *and *E. coli *had a remarkably similar profile that was different from the profile induced by IFN-γ. For the Th2 stimuli, both helminth derived lipid preparations showed a very comparable profile, which resembled the expression profile induced by SEA for most of the genes (fig 3A). However, expression levels in PS pulsed DCs were generally lower than in SEA stimulated DCs which is in accordance with the less pronounced effects on activation of the MAPK by the PS preparations.

**Table 1:** Description of genes

		**Gene**	**Function**	**References**
**Cytokines & chemokines**		IL-12 p35	Together with p40 forms the cytokine IL-12 involved in Th1 polarization	[4,22]
		IL-12 p40	Together with p35 forms the cytokine IL-12 involved in Th1 polarization	[4,22]
		IL-23 p19	Together with p40 forms cytokine IL-23 in DCs which augments Th1 and Th17 responses	[26,32,36]
		TNF-α	General pro-inflammatory cytokine mediating local inflammation. Its expression is dependent on NF-kβ signalling	[17]
		CXCL-10 (IP-10)	Chemotactic factor for T cells. Its expression is dependent on the IFN-responsive gene pathway	[35,45]
		CCL22	Chemokine involved in recruitment of Th2 effector cells	[27]
		TGF-β	Cytokine with anti-inflammatory properties, by inhibiting activity and function of both T cells and DCs	[34,42]
		IL-10	Cytokine that potently suppresses immune responses and in particular DCs and T cell responses, by downregulating pro-inflammatory effectors	[34,37]

**Toll like receptor signalling**		TLR2	Receptor of innate immunity for recognition of mainly lipid containing compounds	[19,43]
		TLR4	Receptor of innate immunity for recognition of LPS	[19,43]
		MyD88	Proximal, most common adaptor of TLR signalling, shared by all TLR except TLR3	[24,43]
		NOD2	Intracellular peptidoglycan receptor implicated in activation of NF-kB but also in inhibition of TLR2 signalling	[31,38,44]
		TRIF (TICAM1)	TLR3 and TLR4 specific adaptor which mediates the MyD88-independent pathway preferentially leading to induction of IFN-responsive genes	[30,46]
		Tollip	Inhibitor of IRAK activity and thereby TLR signalling	[48]
		SOCS-1	Inhibitor of LPS-TLR4 signalling pathway as well as TLR induced JAK/STAT signalling. Potential negative regulator of Th1 responses	[20,47]
		SOCS-3	Inhibitor of JAK/STAT signalling but also positive regulator of APC function by suppression of STAT3, which normally inhibits TLR signalling.	[20,47]

**Modulators of T cell activation & polarization**	**Intracellular**	DUSP2	Phosphatase modulating MAP kinase signalling balance	[33]
		IDO	Enzyme that catabolizes tryptophan to kynurenines, which are able to induce T cell apoptosis and inhibition of proliferation. Expression induced by IFN-γ	[28]
		c-Fos	Transcription factor activated by MAP kinases which induces IL-10 production and is involved in DC mediated Th2/anti-inflammatory responses	[15,18]
	
	**Membrane bound**	PD-L1 (B7-H1)	Costimulatory molecule and ligand for PD-1 on T-cells. It has inhibitory function in T cell proliferation and cytokine production. Might be stimulatory for Th2 response	[25,29,40]
		PD-L2 (B7-DC)	Costimulatory molecule and ligand for PD-1 on T-cells. Reported to have synergic activity with other costimulatory molecules as well as inhibitory activity on T cell activation	[25,29,40]
		LIR-7 (ILT-1)	Receptor with unknown ligand(s) with possible immune suppressive properties, but also implicated in immune activation	[23,41]
		Jagged-2	Ligand for notch-receptor on T cells; influences T cell skewing	[21]
		Delta-4	Ligand for notch-receptor on T cells; influences T cell skewing	[21,39]
		Delta-1	Ligand for notch-receptor on T cells; influences T cell skewing	[21]

Next, we related expression levels of individual genes to the T cell polarizing capacities of the DCs, to identify potential mechanisms through which different pathogen derived compounds induce differential T cell polarization. Members of the IL-12 cytokine family are well known for driving Th1 polarization [1]. Indeed the expression of both IL-12 p40 and p35, but also IL-23 p19 were shown to be upregulated in DCs stimulated with Th1 inducing stimuli and reduced in DCs stimulated with helminth derived compounds (fig. 3A). This was confirmed at the protein level when IL-12 and IL-23 production by DCs were measured by ELISA (Fig 3B, C).

With respect to T cell polarization, other genes of interest are the notch ligand family members delta-1, delta-4 and jagged-2, since expression of delta and jagged on DCs has been associated with induction of Th1 and Th2 responses, respectively [21]. For jagged-2 and delta-1 no significant differences were found between the stimuli (figure 3A). However, delta-4 was upregulated by the bacterial Th1 inducing stimuli, and downregulated by the Th2 inducing lipids. Moreover, expression levels of delta-4 correlated with the IL-4/IFN-γ cytokine ratio produced by the T cells of the stimuli that activate TLR2 (R^2 ^= -0.87, figure 3D). Yet, in SEA and IFN-γ stimulated DCs delta-4 expression was not altered. Therefore, Delta-4 seems to associate with T helper cell polarization only when TLR2 is also engaged.

Conversely, we found higher c-fos mRNA levels in PS and SEA pulsed DCs compared to HKLM and *E. coli *stimulated DCs. c-fos has been shown before to mediate SEA induced repression of IL-12 secretion by DCs [15]. Indeed, correlation analysis revealed that in DCs stimulated with bacterial products or helminth-derived lipids, mRNA levels of c-fos were positively correlated with Th2 induction (R^2 ^= 0.667, fig 3E).

## Discussion

DCs express a range of PRR that allow them to recognize different pathogens and initiate appropriate adaptive immune responses. Pivotal to this process is the proper integration of PRR derived signals into a molecular activation profile of DCs that leads to a particular T cell polarizing capacity. This study demonstrates that combined TLR2 and TLR4 activation in the context of different bacterial and helminth derived extracts can lead to very distinct molecular activation profiles of human DCs which correlate with their T cell polarizing capacity in terms of Th1 and Th2 skewing.

One of the major signalling cascades triggered upon engagement of TLR is the MAPK pathway. Differential activation of MAPK p38 and ERK in DCs has been associated with different level of maturation and cytokine production whereby p38 is thought to be important in mediating DC maturation and pro-inflammatory T cell responses, whereas ERK activation has more often been associated with anti-inflammatory and Th2 responses [49]. Earlier studies in human DCs have primarily focused on the role of different MAPK in DC activation, such as maturation and cytokine production [49]. We extended these studies, by analyzing for the first time the correlation between *p*-ERK/*p*-p38 ratios in human DCs and the degree of skewing of T cell responses by using various Th1 and Th2 inducing pathogen derived extracts. At 20 minutes after stimulation, we observed decreased *p*-ERK/*p*-p38 ratios in the Th1 promoting DCs. Of the two Th1 polarizing agents, *E. coli *induced more p38 activation, compared to HKLM, which is in agreement with the stronger Th1 polarization of the T cells (fig 1D). In contrast, all helminth-derived stimuli increased the *p*-ERK/*p*-p38 ratio in the DCs. Comparison of the MAPK activation profile induced by the helminth-derived lipids with the one induced by SEA, revealed that SEA, like other helminth derived antigens such as LNFPIII [50] and ES-62 [51], induces a higher *p*-ERK/*p*-p38 ratio by increasing activation of ERK. On the other hand, the lipids influenced the *p*-ERK/*p*-p38 ratio by specifically impairing p38 phosphorylation. Thus, although the lipids share the capacity with other helminth antigens described so far to condition DCs for Th2 priming, they appear to achieve this differently exemplified by a different modulation of the MAP kinase signalling pathway. Taken together, the *p*-ERK/*p*-p38 ratio appears to be an important characteristic of antigen presenting cells exposed to pathogen derived compounds that skew responses towards Th2 or Th1.

Comparison of the mRNA expression profiles of TLR activating bacterial and helminth derived compounds revealed that, unlike the Th2 inducing phospholipids, exposure of DCs to Th1 promoting stimuli preferentially led to the induction of the pro-inflammatory cytokines IL-12 and IL-23, both at the mRNA and the protein level. The degree of p38 activation, known to drive pro-inflammatory gene expression by these stimuli, was reflected by the level of expression of these cytokines. The higher expression levels of IL-12 and IL-23 in the *E. coli *and IFN-γ stimulated DCs compared to HKLM pulsed DCs, probably contributes to the stronger Th1 induction seen with the former two stimuli.

While the immunological processes resulting in Th1 polarization have been extensively characterized, it is still poorly understood how exactly Th2 responses are initiated. One of the genes that was found to be positively associated with Th2 inducing DCs was the transcription factor c-fos. c-Fos has been shown to mediate IL-12 suppression in SEA pulsed DCs, which is generally thought to be a prerequisite for Th2 induction [15,18]. In addition, the observation that c-fos mRNA expression levels were strongly correlated with Th2 induction not only for SEA, but also for PS, further supports the notion that this transcription factor plays a role in the promotion of helminth antigen dependent Th2 skewing. However, analysis of c-fos at the protein level revealed that in PS pulsed DCs the increase of c-fos was lower and more transient, compared to SEA stimulated DCs (Everts *et al *unpublished data). Therefore, it remains to be established whether c-fos plays a similar role in PS pulsed DCs as has been shown for DCs modulated by SEA.

Notch ligands have been reported to play a role in Th1/Th2 polarization by DCs [21]. While jagged-2 expression was initially implicated in DC mediated Th2 differentiation [21] more recent studies [52] show that jagged-2 has no role in Th2 induction by SEA activated DCs. Our findings are in accordance with these latter studies, since we did not observe any increased jagged-2 mRNA expression in our helminth derived stimulated DCs. Interestingly, another Notch ligand delta-4 was found to be upregulated in DCs cocultured with bacterial compounds, while helminth derived compounds showed a decreased delta-4 expression. This in agreement with studies that show that delta-4 is involved in Th1 skewing [21], as well as inhibition of Th2 development [53].

Several studies have shown that TLR2 activation alone may lead to different outcomes; Th2 [18,54], Treg [55] as well as Th1 [56]. The variety of outcomes possible in the presence of TLR2 activation have been suggested to be the result of heterodimerization of TLR2 with different receptors, such as TLR1 or TLR6 [57,58], or associations with other receptors including Nod-like receptors and C-type lectins [11,59]. In our study, the compounds used from helminths or bacteria are mixtures of antigens that would be expected to signal via additional receptors besides TLR2. *E. coli *has been shown to activate TLR4 and NOD1 [60,61], whereas resistance to *Listeria *infection was related to the presence of functional NOD2 [62], indicating that this receptor is engaged by HKLM. Relatively little is known about Th2 skewing by the helminth derived compounds, but in a previous study of schistosomal lipids it was shown that TLR2 activation was not needed for Th2, but rather for regulatory responses [6]. Therefore, it is important to study the engagement of additional PRR along with TLR2 and TLR4 to fully understand the mechanisms that play a role in conditioning DCs for priming of Th2 responses [62,63].

## Conclusion

In conclusion, the study presented here indicates that TLR4 ligation on monocyte derived DCs in the context of TLR2 stimulating bacterial or helminth derived extracts leads to profoundly different outcomes in terms of activation or expression of various markers at the level of MAPK phosphorylation, mRNA expression and protein up- or downregulation. We show for the first time in human DCs that the levels of a selected number of molecular markers are strongly correlated with the T cell polarizing capacity of DCs. This not only gives us new insights about the processes involved in Th1 and Th2 polarization but it also suggests that there is a common molecular Th1 and Th2 signature in human DCs that can be used to predict the strength of induced Th skewing in terms of the Th1/Th2 balance.

## Methods

### Antigen preparation

Phosphatidylserine containing preparations (PS) were extracted from 4 gram of *A. lumbricoides *worms (expelled from infected humans) or from schistosomal worms, collected from golden hamsters 45–48 days after infection with *S. mansoni*, as described before [6]. Mass spectrometry was used to confirm the presence and composition of PS species in both lipid preparations, as described before [64]. Schistosomal egg antigen (SEA) was prepared from schistosomal eggs, collected from trypsin treated liver homogenate of the *S. mansoni *infected hamsters. *E. coli *(ATCC 11775) and *L. monocytogenes *(kind gift of J. van Dissel, LUMC, Leiden, The Netherlands) were grown at 37°C for 18 h in Brain Heart Infusion (BHI) bouillon (Biomerieux). Cultures were washed with PBS, quantified, and frozen in aliquots. In addition *L. monocytogenes *was heat inactivated for 2 hours and 45 minutes at 80°C before storage.

### TLR transfected HEK cell activation

HEK-293-CD14, HEK-293-CD14/TLR2 and HEK-293-CD14/TLR4 cells (a gift from Dr. E. Latz, University of Massachusetts) were maintained in DMEM culture medium, supplemented with 10% FCS, 10 μg/ml ciprofloxacin and 5 μg/ml puromycin. For stimulation experiments, cells were seeded at 3.5 × 10^4 ^cells/well in 96-well flatbottom plates and were stimulated the next day. For stimulation of HEK-293-CD14/TLR4 cells, 12.5% supernatant of MD-2 transfected cells was added. IL-8 production was measured in supernatants after 22 hours using a commercial kit (Sanquin, Amsterdam, The Netherlands), by following the manufacturer's recommendations.

### Dendritic cell culture and naïve T cell polarization

Monocytes were isolated and immature DCs were cultured as described before [6]. At day 6 or 7 immature DCs were matured with LPS (ultrapure, *E. coli *0111 B4 strain, Invivogen) (100 ng/ml) in the presence of IFN-γ (1000 U/ml), heat killed *L. monocytogenes *(HKLM; 10^8^/ml), *E. coli *(10^7^/ml), SEA (50 μg/ml), PS lipid extract derived from ascaris worms (an equivalent of 120 μg of worm per ml) or PS lipid extract derived from schistosomal worms (an equivalent of 20 worm-pairs per ml). For RNA isolation, DCs were harvested 16 hours after stimulation, as pilot experiments in our lab indicated that the expression levels of most genes had changed at this time point. DCs were snap-frozen in liquid nitrogen and kept at -80°C until RNA isolation. For measuring cytokine production by DCs and for co-culture with naïve T cells, DCs were matured for 48 hours after stimulation, after which secreted cytokines were measured in the harvested supernatant. Levels of IL12p70 were determined by ELISA using monoclonal antibodies 20C2 and biotinylated mouse-anti-human IL-12 C8.6 (both Becton Dickinson) as coating and detection antibodies, respectively. Levels of IL-23 were determined by ELISA using monoclonal antibodies ebio473p19 and biotinylated mouse-anti-human IL-12 C8.6 (both Becton Dickinson) as coating and detection antibodies, respectively. To determine T cell polarization, 5 × 10^3 ^mature DCs were cocultured with 2 × 10^4 ^naïve T cells that were purified using a human CD4+/CD45RO-column kit (R&D, Minneapolis, MN) in the presence of SEB (100 pg/ml; Sigma) in 96-well flat-bottom plates (Costar). On day 5, rhuIL-2 (10 U/ml, Cetus Corp., Emeryville, CA) was added and the cultures were expanded for another 5–9 days. To measure the frequency of IL-4- and IFN-γ-producing T cells, Th cells were restimulated with PMA and ionomycin in the presence of brefeldinA (all Sigma) during 6 hours and stained with anti-hu-IL-4-PE and anti-hu-IFN-γ-FITC (both BD Biosciences).

### RNA isolation, DNase treatment and cDNA synthesis

RNA isolation was performed using Trizol reagent (Invitrogen, Breda, The Netherlands) according to the manufacturers' instructions, with a minor modification: 3 μl of glycogen (Invitrogen) was added to all samples after they were homogenized in Trizol for a few minutes at room temperature. DNAse treatment and cDNA synthesis were performed following standard procedures.

### Analysis of gene expression levels

Primers and Taqman probes were provided as a Taqman gene expression kit (Applied Biosystems, Foster City, California) or designed using Primer Express (Applied Biosystems) and synthesized by Biolegio (Malden, The Netherlands) and Eurogentec (Seraing, Belgium), respectively (sequences available upon request). Real time qPCR was performed using Eurogentec PCR reagents, in a volume of 25 μl on an ABI PRISM 7700 Sequence Detection System (SDS, Applied Biosystems), using the following program: 10 minutes at 95°C, 40 cycles of 15 seconds denaturation at 95°C and 60 seconds annealing and amplification at 60°C. Results were monitored and analysed with SDS software (Applied Biosystems).

Gene expression was normalized to the housekeeping gene TAF-1 and calculations were performed as described using the 2^-ΔΔC^_T _method [65]. Analysis of the expression of 6 different housekeeping genes in a subset of the samples indicated that TAF-1 was the most stable housekeeping gene in our samples upon stimulation. Spotfire software  was used to generate a heatmap and perform hierarchical clustering of the genes.

### MAPK activation analysis

20 and 60 minutes after stimulation of immature DCs (day 6), cells were fixed for 10 minutes with 4% ultrapure formaldehyde (Polysciences) directly in the plate. Cells were harvested and washed twice in PBS/0.5% BSA. Subsequently, the DCs were permeabilized in 700 μl ice-cold 90% methanol in PBS in and left on ice for 30 minutes. Following two wash steps in PBS/0.5%BSA intracellular staining was performed for 2 hours at room temperature in the dark with anti-phospho-p44/42 MAPK AF-488 (T202/Y204) and anti-phospho-p38 MAPK AF-647 (T180/Y182), (Cell Signalling Technology). After one wash in PBS/0.5%BSA MAPK activation was determined by flow cytometry using a Becton Dickinson FACSCalibur flowcytometer (BD Biosciences) and analysed using FlowJo analysis software (Tree Star).

### Statistical analysis

Data were analysed using SPSS (v14.0) and GraphPad Prism4. Differences among stimuli were analysed by a Mann-Whitney test. Differences relative to LPS stimulation were determined using a one sample t-test. Correlations between expression of genes and/or T-cell responses were calculated by a two-tailed Spearman's-rho test. Differences were considered significant when P-values were below 0.05.

## Authors' contributions

ER and BE participated in the design of the study, drafted the manuscript, and together with MP performed and analysed the dendritic cell experiments. KR, JH and AT were involved in isolation and characterization of the helminth derived lipids. DK, FH and MY were involved in designing and coordination of the study, and interpretation of the data. All authors were involved in revising the manuscript and all authors read and approved the final manuscript.
